# The Institutional Worker

**Published:** 1918-06-15

**Authors:** 


### INSTITUTIONAL WORKER

**Being a Special Supplement to "The Hospital"**

### OUR BUREAU OF INFORMATION.

**URGENT NOTICE.**

To ensure a copy of The Hospital regularly, readers mu t place a definite order with their Newsagent TO-DAY. After the middle of June, in consequence of the new Paper Restrictions Order, it will not be possible for Newsagents to stock copi s for chance customers.

DO NOT DELAY. ORDER

"THE HOSPITAL" TO-DAY

and obtain your copy regularly EVERY THURSDAY.

### A Register of Nurses' Ward Work.

### Answer to April Question.

**The question was:**

**How should a book be ruled to give at a glance a record of nurses' different duties in male, female and children's wards, and time spent at classes, etc.? The hospital is a training-school, and nurses take day and night duty. There are three floors for male patients, two for female patients, and a children's block.**

**By A. M.**

I would suggest that a very wide book with an alphabetical index be procured, and every particular relating to a nurse written on one page.

The following particulars would occupy the five top lines of both left and right hand pages.

Name.........Age.........Date of Birth.........Religion.........
Entered for training.........
Previous nursing experience.........
Other details.........
Home address.........

Ruling for Left-hand Page.

| Ward | No. of Weeks | From | To | Duty | Remarks |
|---|---|---|---|---|---|
| Male Floor No. 1 ... ... | 12 | 15/10/17 | 31/12/17 | Junior probationer | Good report |

Then follow in the column headed "Ward" the names of the various wards or departments (Male Floor No. 2; Male Floor No. 3; Female Floor No. 1; Female Floor No. 2; Children's Block; Theatre; Out-patients and Casualty Department; Electrical and Massage Department; Special), each on a separate line, so that similar particulars to those given as an example above may be entered.

Ruling for Right-hand Page.

| Subject | Instructor | No. of Classes | From | To | No. of Attendances | Result of Exam. (Max. 50) | Remarks |
|---|---|---|---|---|---|---|---|
| Elementary Nursing | Matron | 12 | 7/1/18 | 25/3/18 | 12 | 40 marks | Good paper |

Then follow in column headed "Subject" the names of the subjects of classes, allowing a separate line for each, and entering against each the particulars as under Elementary Nursing (Bandaging, Anatomy, Physiology, Medical Nursing, Surgical Nursing, Special, Sick Cookery).

HOLIDAYS.

| Year | No. of Weeks | From | To | Remarks |
|---|---|---|---|---|
| 1st year ... | 3 | | | |
| 2nd year ... | | | | |
| 3rd year ... | | | | |
| 4th year ... | | | | |

SICKNESS.

| Disease | O Duy | From | To | Sick Leave | From | To | Remarks |
|---|---|---|---|---|---|---|---|
| | | | | | | | |

All headings, also periods of night duty, to be written in red or other coloured ink.

### JUNE QUESTIONS.

**1. Equipping an X-ray Department.**

Give a list of equipment for an X-Ray, Electrical, and Massage Department in a hospital of 180 beds.

**2. Restrainer for Diphtheria Case.**

Describe a simple, washable restrainer, suitable for helping to keep flat in bed a young child suffering from diphtheria. Such a patient, after the first week or so, does not feel ill, and it is almost impossible to keep a child flat in bed without some kind of restrainer.

### RULES.

The following rules must be observed:—

1. Contributions must be written on one side of the paper. Brevity and terseness are desirable features. The MSS. must bear the name and address of the sender and be accompanied by coupon to be cut from the back cover (inside page) of the current issue of THE HOSPITAL. A pseudonym must be chosen if the name is not to be published.

2. Contributions must be addressed to the Editor of THE HOSPITAL, Institutional Worker Supplement, 28 & 29 Southampton Street, Strand, London, W.C. 2, should reach him before the end of the current month, and be marked in the left-hand corner "Facts and Figures."

**A minimum payment of Five Shillings will be made for each published answer.**

### QUESTIONS INVITED.

In connection with our Question Box, we offer every month five shillings each for the two best questions which are sent in for consideration. The questions may be on any institutional subject and concern any department, from the secretary's to the porter's, or the matron's to the domestic staff; the one essential is that they must be practical and deal with points that have a definite relation to hospital or institutional administration, upkeep, finance, or management. Points relating to artificers' work, housekeeping, the laundry, out-patients, and other practical matters will be welcome. It is hoped that thus, when difficult or doubtful points arise in the course of their work, institutional workers will be encouraged to put them in the form of a question and send them to the Editor, THE HOSPITAL Bureau of Information, 28 & 29 Southampton Street, Strand, W.C. 2, marked "Question Box."

The best questions will be published in due course and our readers will be given the opportunity of answering them. Thus all institutional workers may be able to cooperate with the view of helping the work and smoothing the difficulties of each other. In short, we want the Question Box of the *Institutional Worker* to be freely used by every worker habitually.

### ENQUIRIES AND ANSWERS.

### *THE SICK AND IN NEED.*

### Royal West of England Sanatorium—Terms of Admission.

THE terms of admission to the Royal West of England Sanatorium, Weston-super-Mare, are by payment of a subscription of £1 1s. and a weekly payment of 5s. for a fortnight's residence. Upon the receipt of £1 11s. a recommendation note will be sent which must be filled in and signed by the patient's medical attendant. Patients are admitted on Mondays, but no vacancy can be promised in advance nor until the payment has been received. All correspondence should be addressed to the Secretary-Superintendent. A stamp should be enclosed for reply.

### Case of Spinal Curvature.

WE suggest that you apply to the Secretary, Lord Mayor Treloar's Cripples' Hospital, 61 Moorgate Street, E.C., for admission to the hospital, which is situated at Alton, Hants. You could be quite sure of the child receiving the very best care and attention at this institution.—VISITOR.

### *EMPLOYMENT AND TRAINING.*

### Private Nursing Institutions in Bristol.

AMONGST the private nursing institutions in Bristol are: The Bristol General Hospital Private Nursing Institution; Bristol Hospital for Sick Children and Women's Private Nursing Staff; Bristol Royal Infirmary Private Nursing Staff. (All these institutions train their own nurses.) Also the Bristol Nurses' Institution and Nursing Home, 3 and 4 Chesterfield Place, Clifton, Bristol. You will find particulars of these institutions in "How to Succeed as a Trained Nurse," published by The Scientific Press, Ltd., 28 and 29 Southampton Street, Strand, W.C. 2, price 2s. 6d. net.—KORA.

### Zander Mechanotherapy.

YOU might possibly obtain instruction in Zander mechanotherapy by applying to either of the following:—Dr. Hamel, Zander Institute, 1 Stratford Place, W.; the Edgar Allen Institute, Sheffield; or the Zander Institute, Bath. At any rate, if you are unable to make arrangements for a course of instruction, you may possibly obtain advice upon the matter—K. T.

### Post as Masseuse in Military Hospital.

APPLICATIONS for posts in military hospitals in the London district should be made to the D.D.M.S., London District, Headquarters, Horse Guards, S.W. It is necessary that applicants should be members of the Almeric Paget Corps.—C. I. P.

### *MISCELLANEOUS.*

### Book on Medical Electricity.

PROBABLY "Medical Electricity," by Dr. Lewis Jones and published by Messrs. H. K. Lewis and Co., Ltd., might meet your requirements. The price is 15s. net. The sections on the treatment of nerve injuries are both clear and practical, and especially suitable to those engaged in the treatment of the wounded.—THEODORE.

### Door-Holders.

A USEFUL door-holder is made by Messrs. Nettlefold and Sons, Ltd., of 54 High Holborn, W.C. It consists of a lever swinging on a strong pin attached to a plate which is screwed to the bottom of the door. At the end of the lever there is a cushion of rubber which engages the floor when the lever is pressed over. The lever can be actuated by the foot, and is kept in position by a spring.—G. M.

### Repair of Cystoscope.

YES, it is possible to get your cystoscope repaired, but we fear that you will have to be patient. In pre-war days cystoscopes came from Austria and Germany, and were, as a rule, sent back for repair. There are very few artisans in this country who have yet grasped the technique of this delicate work, and some of the earlier types of instruments present many difficulties, for they consist of a single tube into which is fitted perhaps as many as ten lenses and reflectors. It will therefore be realised that the displacement of one lens may cause much work in the repair. The more modern instruments are built in sections, and are more easily dealt with. Among other manufacturers, Messrs. Allen and Hanburys, Ltd., of Wigmore Street, W., are carrying out this work, and we suggest that you apply to them. We doubt whether you can obtain an American cystoscope at this time.—WORRIED.

### *THE HOUSEKEEPERS' DEPARTMENT.*

### Eggs and Spinach.

A LIGHT supper dish and one which is fairly economical in these days is eggs and spinach. Prepare and cook the spinach in the usual way, but, in addition to the small piece of margarine or butter added, pour over some good gravy. Boil this for ten or fifteen minutes very quickly and add pepper, salt, and nutmeg. Poach the necessary number of eggs, trim them neatly, and place on the spinach.—T. A. T.

### Dish of Mashed Potatoes.

To 2 lb. of potatoes you will require 1 oz. margarine or butter and $\frac{1}{4}$ to $\frac{1}{2}$ pint milk, and seasoning. Boil or steam the potatoes, and pass them through a wire sieve. Melt the butter, add the milk, and stir in the potatoes when the milk is boiling; beat the whole very thoroughly; add the seasoning. This should be served very hot, of course.—P. S.

